# Benchmarking newborn care quality in Ghana: Evidence from structured observations of clinical practice against WHO quality standards

**DOI:** 10.1371/journal.pone.0350931

**Published:** 2026-06-11

**Authors:** Solomon Mohammed Salia, Billie de Haas, Jelle Stekelenburg, Robert Kaba Alhassan

**Affiliations:** 1 Department of Nursing, School of Nursing and Midwifery, University of Health and Allied Sciences, Ho, Ghana; 2 Department of Health Sciences, Global Health Unit, University Medical Centre Groningen/University of Groningen, Groningen, The Netherlands; 3 Faculty of Spatial Sciences, Population Research Centre, University of Groningen, Groningen, The Netherlands; 4 Frisius Medical Centre, Department of Obstetrics and Gynaecology, Leeuwarden, The Netherlands; 5 School of Health Science, University of Dundee, Dundee, Scotland, United Kingdom; 6 Centre for Health Policy and Implementation Research. Institute of Health Research, University of Health and Allied Sciences, Ho, Ghana; Chinese Academy of Medical Sciences and Peking Union Medical College, CHINA

## Abstract

**Introduction:**

The neonatal mortality rate in Ghana remains above the global average, which is far from meeting SDG 3.2. Notably, institutional neonatal deaths are on the increase, potentially due to healthcare professionals’ poor adherence to quality newborn care guidelines. This study assessed the adherence of clinical healthcare professionals to guidelines on quality newborn care in Ghana.

**Methods:**

This observational study collected data from 158 healthcare professionals across six health facilities in Ghana. A data collection tool was developed based on the *WHO standard for improving quality of care for small and sick newborns and the Ghana Health Services Standards for Newborn Health Services*. The Mann-Whitney U test was used to measure differences in categorical data, while logistic regression models were employed to analyse the association between dependent and independent variables at a 95% confidence level. Six facility-level discussions with the observed healthcare professionals were held to validate and interpret the quantitative findings.

**Results:**

Overall, only 31 (20%) of healthcare professionals demonstrated good adherence to infection prevention and control (IPC), while 73 (46%) adhered poorly. Moderate adherence levels were seen in essential care for every newborn (ECEN) at 53% and respectful maternal and newborn care at 51%. During validation discussions, healthcare professionals mentioned poorly located handwashing materials, parental preference for methylated spirit over chlorhexidine for umbilical cord care, and lack of protocols for pain assessment as key barriers to adherence.

**Conclusion:**

The study findings revealed low adherence levels to quality newborn care practices, especially in IPC practices. Moderate adherence levels were found in ECEN and respectful maternal and newborn care. These findings highlight the need to improve adherence with quality newborn care standards in Ghana. The Institutional Care and Family Health Divisions of the Ghana Health Service should integrate these findings into ongoing quality improvement efforts to achieve better neonatal outcomes.

## Introduction

In 2022, approximately 2.3 million newborns died worldwide, with 75% of these deaths occurring within the first week of life [1]. Despite substantial efforts to reduce neonatal mortality (NM), it remains alarmingly high, especially in sub-Saharan Africa (SSA), where the neonatal mortality rate (NMR) is currently 27 deaths per 1,000 live births compared to the global rate of 17 deaths per 1,000 live births [2]. Newborns in SSA are 11 times more likely to die than those in many developed countries, such as Australia and New Zealand [2]. Ghana, like many SSA nations, continues to struggle with high neonatal mortality. The country’s NMR currently stands at 17 deaths per 1,000 live births [3]. The leading causes of neonatal death in Ghana include complications of prematurity, birth complications (birth injuries/asphyxia), newborn infections, low birth weight, neonatal jaundice, and congenital malformations [4–6]. Most of these causes are preventable.

Despite significant efforts to achieve the Millennium Development Goals (MDGs), Ghana did not meet MDG 4 by its 2015 deadline, as progress stagnated, and neonatal mortality rates temporarily increased, reaching an estimated 28.3 [7]. However, the country has since made notable strides in lowering the NMR through locally tailored interventions while supporting global initiatives. To address the rising neonatal mortality rates, Ghana has implemented several national strategic interventions, including the National Health Insurance Scheme (NHIS), the Free Maternal Healthcare Policy (FMHCP), and the establishment of neonatal intensive care units in hospitals nationwide. Additionally, Ghana introduced the Ghana National Newborn Health Strategy and Action Plan (2014–2018) to speed up progress in reducing preventable neonatal deaths [8], which was later revised and extended to 2023 [9]. Investments in health infrastructure, such as the “Agenda 111” initiative [10] and the national ambulance expansion programme called “One Constituency, One Ambulance" [11], further strengthened gains in maternal and newborn health. The combined effect of these efforts led to substantial increases in antenatal care (98%), postnatal care (87%), and facility-based deliveries (88%), helping to reduce NMR [3], in line with Sustainable Development Goal (SDG) 3.2, to end preventable deaths of newborns and children under five by 2030. Though these interventions are timely, concerns remain. For example, the harmonised health facility assessment findings in 2023 showed that only a small portion (20%) of health facilities in Ghana provide full comprehensive emergency obstetric and newborn care (CEmONC), and roughly 29% of these facilities have guidelines on essential newborn care practices, with just 45% of staff trained in essential newborn care [12].

While these national initiatives have created a foundation for improved newborn care, ensuring consistent quality across facilities requires alignment with international standards. The WHO developed a comprehensive quality framework, known as *Standards for improving the quality of care for small and sick newborns in health facilities,* to enhance the quality of newborn care. This framework comprises three domains: *provision of care*, *experience of care*, and *outcomes*, each containing specific standards, quality statements, and measures aimed at promoting high-quality practices among healthcare professionals. Overall, the framework includes eight standards and 80 quality statements [13]. Responding to this call, Ghana’s Ghana Health Service (GHS) developed and implemented the *Standards for Newborn Health Services* in 2020 to guide consistent and standardised evidence-based care across all levels of the health system. Similar to the WHO quality of care (QoC) framework, the GHS standards specify 20 key standards covering respectful maternal and newborn care, routine newborn care, infection prevention and control, and management of complications [14]. Since 2017, Ghana has been a member of the Quality of Care (QoC) Network for Maternal, Newborn, and Child Health (MNCH), which promotes standards-based care and ongoing quality improvement [15]. In 2018, Ghana adopted, adapted, and implemented the WHO standards to improve maternal and newborn health care throughout the country [15]. With support from UNICEF and other partners, this initiative was expanded to all 16 regions, covering 186 QoC/MNCH facilities. It facilitated training for healthcare professionals (HCPs) across various levels, focusing on essential care for small and sick newborns, kangaroo mother care, infection prevention, and more. The initiative also established Point of Care Quality Improvement in all health facilities nationwide.

Despite these efforts, a critical gap remains in understanding how healthcare professionals apply these standard care guidelines in real-time clinical settings. Several studies have examined facility readiness and service availability to improve newborn care quality in Ghana [16–18], but, to our knowledge, none have specifically examined adherence to WHO quality standards specifically for newborn care. The current study is based on the WHO framework *standards for improving quality of care for small and sick newborns in health facilities.* Specifically, the *provision of care* and the *experience of care* domains, including three quality standards aligned with these domains, are utilised: Standard 1 (provision of evidence-based routine care and management of neonatal complications); Standard 4 (effective communication and meaningful participation); Standard 5 (respect and preservation of dignity). Given the established link between adherence to quality standard guidelines and neonatal outcomes [19–21], evaluating clinical practices in real-time against WHO benchmarks is timely. Such benchmarking can offer actionable insights for improving care delivery, training, and accountability within Ghana’s health system. Using observation provides a strong method to capture actual care practices, revealing not only the services provided but also how they are implemented. Therefore, this study examined the quality of newborn care in Ghana by comparing observed clinical practices with WHO standards across the northern, middle, and coastal belts. Additionally, facility-level discussions with healthcare providers and relevant stakeholders were conducted to validate and enrich the understanding of the findings.

## Materials and methods

### Ethical considerations

Ethical approval was obtained from the Ghana Health Service Ethics Review Committee (GHS-ERC-GHS-ERC 009/04/23) and the Komfo Anokye Teaching Hospital Institutional Review Board (KATH IRB): KATH IRB/AP/003/24. Written permission was secured at the facility level. To prevent potential discomfort complaints from participants during observation, researchers guaranteed that voluntary written informed consent was obtained before starting the study. This consent explained the study’s nature, procedures, purpose, and all ethical considerations. Participants were informed that while they would not receive monetary benefits, the data collected would be published for a broader readership, potentially enhancing newborn care practices in health facilities. They were also reminded of their right to participate or withdraw from the study at any time without repercussion. Additionally, participants were assured that no video recordings of their care practices would occur. Researchers strictly adhered to all facility rules and regulations to ensure smooth data collection. During observations, the principal investigator (PI) ensured that research assistants behaved ethically and upheld data collection standards. Caregivers were not directly informed about the observations, as they were not involved, but were informed during the process and cooperated.

### Study design

This mixed-method observational study gathered data from clinical healthcare professionals on their adherence to quality newborn care practices across six Ghanaian healthcare facilities. The observational design employed quantitative ethnographic methods. Real-time behaviours of healthcare professionals were recorded using a checklist for statistical analysis. This approach enabled assessment of the quality of care (QoC) and adherence to standard guidelines [22]. The qualitative component obtained valuable data during validation discussions to confirm study findings. This study is part of a larger initiative aimed at improving newborn care quality to meet SDG 3.2. The study was modelled using the WHO standards for improving the quality of care for small and sick newborns [13], which is also in line with the GHS Standards for Newborn Health Services in Ghana [14]. The observational study followed the Strengthening Reporting of Observational Studies in Epidemiology (STROBE) guidelines [23]. See details of the STROBE checklist as Supporting File 1 (S1 File).

### Study site

This study was conducted in six health facilities in the 16 administrative regions of Ghana. The 2021 national housing and population census puts Ghana’s population at 30.8 million. Children (0–14 years) and young adults (15–25 years) make up 35% and 38% of the population, respectively [24]. The total fertility rate (TFR) was 3.9 in 2022, down from 6.4 in 1988, with a median age at first birth of 22.1 years [3]. Ghana was purposefully divided into three geographical belts: Northern (northern, northeast, savanna, upper east, and upper west regions); Middle (Ashanti, eastern, Bono, Bono East, Ahafo, Oti, and Western North); and Coastal (Greater Accra, Central, Volta, and Western). Two health facilities, anonymised with letters, were selected from each zone: from the northern zone (Facility A and B), the middle zone (Facility C and D), and the coastal zone (Facility E and F). Facilities A, C, and E are tertiary, while B, D, and F are primary and secondary, respectively. All the selected facilities are credentialed by the National Health Insurance Authority (NHIA) and accredited by the Health Facilities Regulatory Agency (HeFRA) [25, 26]. Further details on the study site are published in a previous study by Salia et al. [27].

### Study population

Participants primarily included nurses, midwives of all cadres, and medical practitioners (doctors and physician assistants) providing direct neonatal care in the NICUs of these facilities. In total, 200 HCPs were identified across the facilities. In Ghana, the curriculum for the training of nurses, midwives, and medical practitioners includes subjects or courses on newborn care, where they acquire knowledge and skills to provide essential newborn care practices (ENCP), including neonatal resuscitation [28,29]. Those with at least three months of NICU experience for nurses/midwives or at least one month for medical practitioners were eligible. Other NICU staff not involved in clinical care were excluded. Find further details in Salia et al publication [27].

### Sampling technique and sample size determination

**Fig 1:**
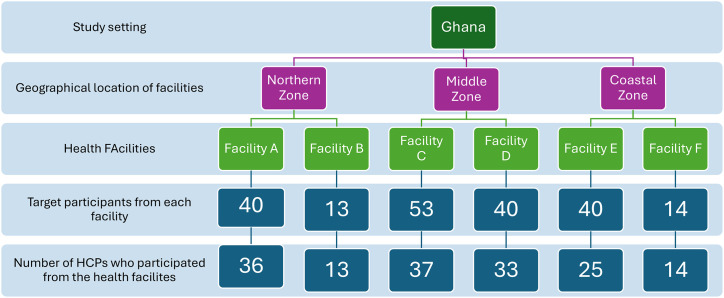
Summary of Participants’ Sampling and Recruitment.

Multistage sampling was employed. Firstly, tertiary facilities were selected purposively, while secondary and primary facilities were sampled using simple random sampling. Due to the small number of HCPs who were present at the NICUs, the researchers employed a census method of sampling once they provided consent. Therefore, 158 out of the 200 HCPs consented and participated. The participant selection and facility sampling are detailed in Fig. 1.

### Data collection tool

The checklist for data collection was developed from quality statements adapted from the WHO newborn care standards. Similar published studies from Namibia [30], Ethiopia [31], and Italy [32] have previously utilised the WHO quality of care standard frameworks. This study tool included quality statements from the *provision of care* and *experience of care* domains, utilising three main standards as mentioned previously. Standard 1 was further divided into infection prevention/control (IPC) and essential care for every newborn, while Standards 4 and 5 were combined as respectful maternal and newborn care. Full details of the standards and quality statements that guided the study tool development are found in Supporting File (S1 Table). A detailed description of the study tool is published in the Salia et al. study [27].

### Selecting and training research assistants for data collection

One trained research assistant (RA) from each facility was recruited and trained to conduct the observations with the principal investigator (first author). Selecting the RAs from the data collection facilities was to help minimise the Hawthorne effect in observational studies, where people tend to change their attitude or behaviour towards a particular act when they are observed [33]. The assumption is that the HCPs are less likely to change their usual care practices when observed by people they are familiar with, as opposed to strangers they do not know. The RAs were selected because of their knowledge of newborn care practices. The training sessions were organised by the PI and facilitated by the PI and a neonatal nurse specialist, focusing on how to collect observational data while ensuring data quality and consistency.

### Data collection methods and procedures

Data collection commenced in August 2023 and ended in April 2024. Data was mainly collected through structured observations on a facility basis by the PI and RAs. This method was participatory but non-intrusive, where the observers attended to some commands whenever the HCPs instructed, without interfering with the actual care practices of the HCPs [33]. The PI and RAs independently observed each participant at a time. In this case, each observer independently recorded what was observed without any influence from the other. At the end of each observation, the recordings were compared for cross-validation of the observations. Where disagreement occurred, the observers discussed clarifying the recordings to avoid biases. The information collected initially included completing a self-administered questionnaire containing demographic, individual, and facility-level factors. The observers then observed the HCPs using a checklist as they provided care to the babies in the NICUs. Data were collected daily from morning to evening until all items on the checklist were completed. Each day after data collection, the PI collected all completed questionnaires from the participants and took responsibility for all observational checklists until the items were completely observed.

### Quality control measures

The researchers ensured that data collection methods were consistent throughout the observation period to avoid biases and maintain data integrity. Also, rigorous ethical processes ensured the study was registered with internationally recognised ethical review boards (Ghana Health Service Ethical Review Committee [GHS-ERC] and the Komfo-Anokye Teaching Hospital Institutional Review Board). These processes were followed to safeguard participants’ rights, privacy, and safety during data collection without undue discomfort. To increase validity and reliability, neonatal and paediatric clinical specialists and the three co-authors reviewed the checklist. Finally, the research assistants received training on the data collection tool and the procedure for observations to maintain consistency in data collection and integrity of the results. The tool yielded scale reliability for IPC, essential care, and respectful maternal-newborn as 0.70, 0.70, and 0.65, respectively, which are generally acceptable values in health research [34, 35].

### Facility-Level data collection and validation

To validate and better understand the quantified observation findings, the observed HCPs and other relevant stakeholders participated in data validation sessions to discuss the preliminary findings. About 80 people participated in this dissemination exercise in all the facilities, with the highest attendance at Facility A. During this validation process, the PI presented the findings and led the subsequent discussions. Responses of participants were audio recorded for verbatim transcription. These data were deductively coded using the items from the observation checklist.

### Data analyses

**Fig 2:**
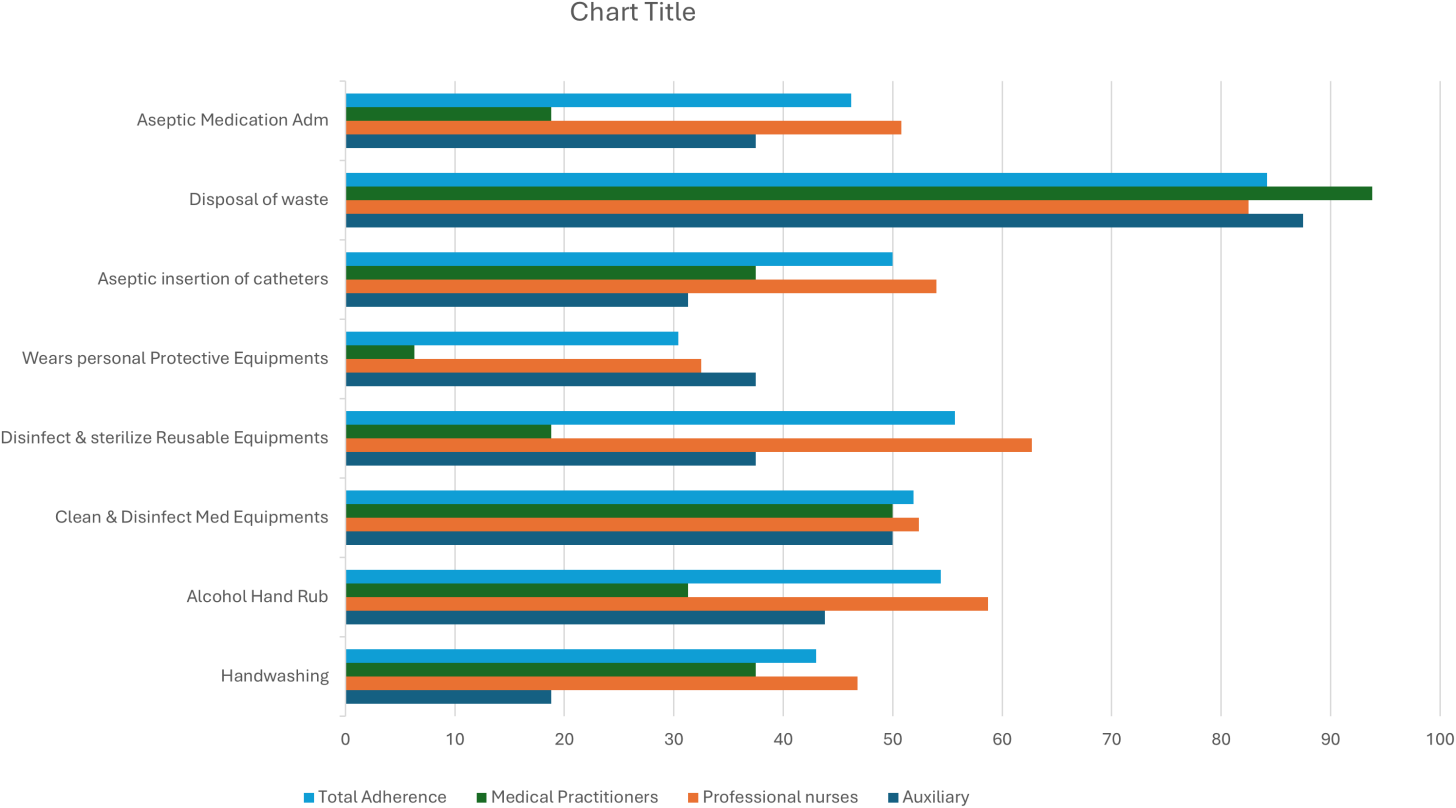
Healthcare professionals’ adherence to quality infection prevention and control measures.

**Fig 3:**
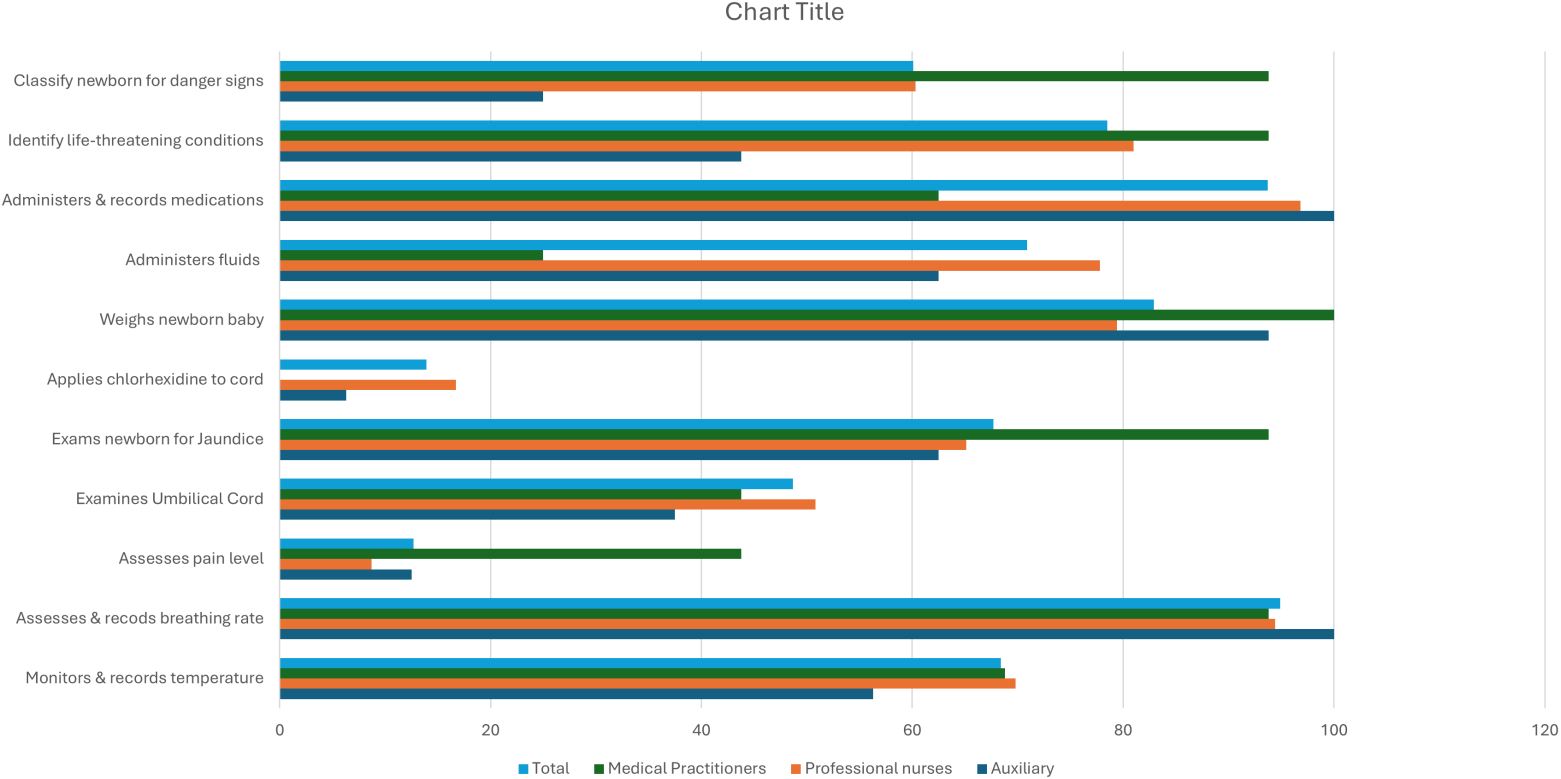
Healthcare professionals’ adherence to quality essential care for every baby.

**Fig 4:**
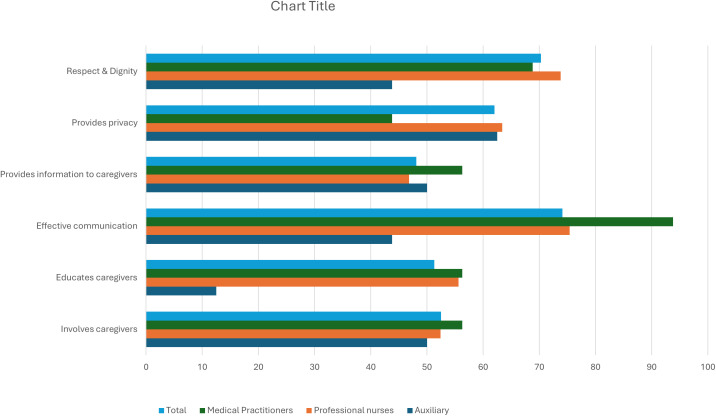
Healthcare professionals’ adherence to quality respectful maternal and newborn care.

The quantitative data were managed and analysed with STATA version 14.0. The independent variables (demographic characteristics, individual factors, and facility-level factors) were analysed using frequencies and percentages and presented in Tables 1 and 2. The main outcome variable was quality newborn care, which was decomposed into infection prevention and control, essential care for every newborn, and respectful maternal and newborn care. Items in each of the outcome variables were scored on a four-point Likert scale which were subsequently dichotomised into “not adherent” as 1 (combining scores of 1 and 2) and “adherent” as 2 (combining scores of 3 and 4). The findings are presented as frequencies and percentages in Table 3. However, the mean scores of the level of adherence to quality newborn care practices (adherent and not adherent) were calculated on the original four-point Likert scale as presented in Table 3. Cross-tabulation analyses were also done to determine the adherence levels between the dependent and independent variables and presented in Figs 2–4.

**Table 1:** Socio-demographic characteristics and individual-level factors of participants.

Variable	Frequency (N)	Percentage (%)
**Participants’ ages (years)**		
25-31 years	99	63
32-37 years	36	23
38-44 years	23	15
**Sex**		
Male	47	30
Female	111	70
**Marital Status**		
Single	78	49
Married	80	51
**Ethnicity**		
Dagaare/Waali	25	16
Dagbani	24	15
Akan	82	52
Ewe	27	17
**Religion**		
Islam	29	18
Christianity	129	82
**Professional Educational Qualification**		
Certificate	16	10
Diploma	47	30
BSc/BA/BEd	83	53
Masters/Membership/Fellowship	12	8
**Professional Category**	N = 158	
Auxiliary nurses	16	10
Professional nurses	126	79
Medical Practitioners	16	10
Rank (Auxiliary)	N = 16	
Enroll nurse	7	4
Senior Enroll nurse	9	6
**Rank (Professional Nurse)**	N = 126	
Staff nurse	24	15
Senior staff nurse/Senior staff midwife	25	16
Nursing/midwifery Officer	45	29
Senior nursing/midwifery Officer	23	15
Principal Nursing/Midwifery Officer	9	6
**Rank (Medical Professional)**		
Junior Medical Practitioners	10	6
Senior Medical Practitioners	6	4
**Number of years of work experience**		
Less than 5 years	87	55
More than 5 years	71	45
**Number of years of work experience in the current ward**		
Less than 5 years	125	79
More than 5 years	33	21
**Received training on ENCP**	N = 158	
Yes	154	98
No	4	3
**Period since last training**	N = 154	
Less than 6 months	97	61
6 months and above	57	36

**Source of data: Field data (2024).**

***Legend:*** *BSc: Bachelor of Science; BA: Bachelor of Arts; BEd: Bachelor of Education; ENCP: Essential Newborn Care Practices.*

**Table 2:** Facility Level Factors.

Variables	Frequency (N)	Percentage (%)
**Level of facility**		
Primary	13	8
Secondary	46	29
Tertiary	99	63
**Location of facility**		
Northern Zone	49	31
Middle Zone	70	44
Southern/Coastal Zone	39	25
**Facility ownership**		
Private Non-profit	33	21
Public	125	79
**The health facility organised training on ENCP**		
No	10	6
Yes	148	94
**Attended training at the facility on ENCP**		
No	21	13
Yes	138	87
**Received regular monitoring and supervision on newborn care at the facility**		
No	28	18
Yes	130	82
**Availability of up-to-date newborn care guidelines**		
No	25	16
Yes	133	84
**Availability of logistics and supplies in sufficient quantities**		
No	89	56
Yes	69	44
**Availability of essential medicines in sufficient quantities**		
No	64	41
Yes	94	60
**Availability of medical equipment at neonatal intensive care unit (NICU)**		
No	50	32
Yes	108	68
**Facility organised clinical conferences/mortality meetings**		
No	15	10
Yes	143	91
**Attended the clinical conferences/mortality meetings organised by facility**		
No	82	52
Yes	76	48
**Effective referral system**		
No	7	4
Yes	151	96
**The neonatal intensive care unit is adequately staffed**		
No	129	82
Yes	29	18
**The neonatal intensive care unit has adequate hand hygiene facilities**		
No	23	15
Yes	135	85

Source: Field data (2023–2024).

Legend: ENCP = Essential Newborn Care Practices; NICU = Neonatal Intensive Care Unit.

**Table 3:** Healthcare professionals’ adherence to quality newborn care statement measures adapted from the WHO quality of care for small and sick newborns framework.

				Adherence Level (%)		
**Quality Domain**	**Quality Standard**	**Substandard**	**Quality Statements (Variables)**	**Not Adherent**	**Adherent**	**Mean scores of Adherence**
PROVISION CARE	**Standard 1:** Every small and sick newborn receives evidence-based routine care and management of complications	INFECTION PREVENTION AND CONTROL MEASURES	1. Adopts standard guidelines on hand hygiene to wash hands with soap and water	57.0	43	2.38
			2. Adopts standard guidelines on hand hygiene to perform alcohol hand rub	46	54	2.47
			3. Adopts standard guidelines to clean and disinfect medical equipment	48	52	2.33
			4. Adopts standard guidelines to clean medical instruments before use	44	56	2.40
			5. Wears appropriate personal protective equipment (PPEs) during newborn care	70	30	2.16
			6. Adopts standard guidelines and procedures to insert and maintain indwelling catheters aseptically	50.0	50	2.44
			7. Adopts standard guidelines to dispose of medical waste and sharps	16	84	2.84
		ESSENTIAL ROUTINE CARE FOR EVERY BABY	1. Uses standard procedures to monitor and record newborn’s axillary temperature	32	68	2.67
			2. Assesses and records newborn’s breathing rate	5	95	2.90
			3. Uses appropriate pain assessment tools to assess newborn pain	87	13	1.63
			4. Examines newborn’s eyes, skin, and mucus membranes for neonatal jaundice	32	68	2.54
			5. Examines the newborn’s umbilical cord and eyes for infection	51	49	1.99
**Continuation** PROVISION CARE		ESSENTIAL CARE FOR EVERY BABY	6. Applies chlorhexidine gluconate 4% or 7.1% to the umbilical cord	86	14	1.38
			7. Weighs babies daily using a calibrated weighing scale	17	83	2.90
			8. Administers prescribed intravenous infusions and/or parenteral nutrition through infusion pumps and DOSI-FLOW.	29	71	2.70
			9. Appropriately records medications and infusions after administration	6	94	2.94
			10. Identifies newborns with life-threatening conditions	22	79	2.73
			11. Classifies newborns with neonatal danger signs	40	60	2.47
EXPERIENCE OF CARE	**Standard 4:** Effective communication and meaningful caregivers’ participation	Meaningful caregiver participation	1. Identifies caregivers and involves them in the decision-making of the care of their babies	48	53	2.15
			2. Educates caregivers on newborn care practices during admission or on discharge	49	51	2.25
			3. Provides information about the health of newborns to their caregivers	52	48	2.03
			4. Demonstrates effective communication skills towards caregivers of newborns	26	74	2.90
	**Standard 5:** Respect and Preservation of Dignity	Respectful maternal and newborn care	1. Demonstrates respect for caregivers and their newborns during care	30	70	2.49
			2. Provides privacy to caregivers and their newborns during care	38	62	2.44

Source: Field data (2023–2024).

Legend: All mean scores in Table 3 were calculated on a four-point Likert scale; highest mean score = 4.0; lowest mean score = 1.0.

**Fig 5:**
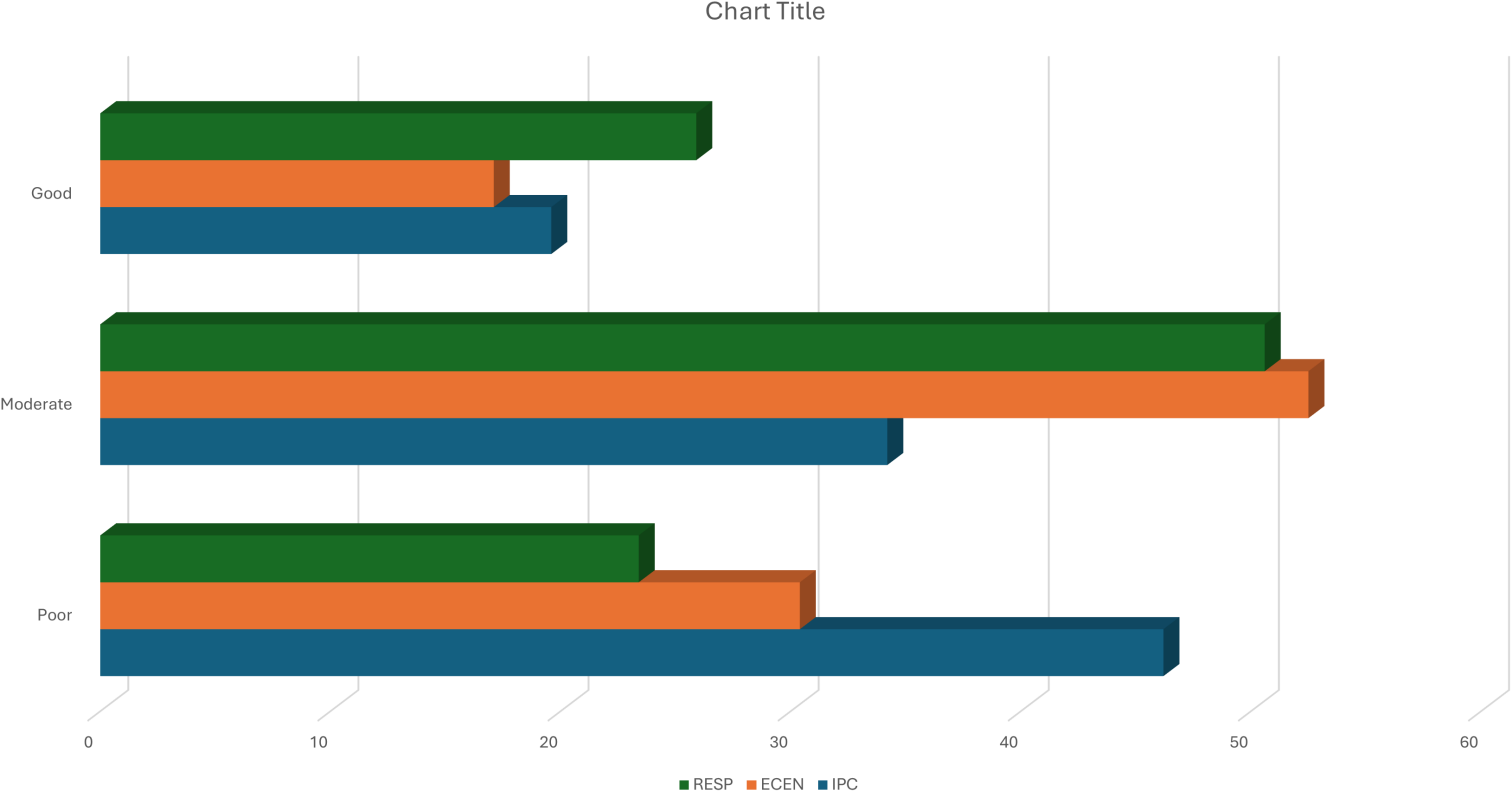
Overall adherence level to quality newborn care practices.

Nonparametric methods, specifically the Mann-Whitney U test, was used to compare differences in mean scores between professional categorisations and number of years of work experience with the decomposed outcome variables at a confidence interval of 95% with a significance of less than 0.05. The differences in mean scores showed adherence to quality newborn care. The total raw score of the items in the decomposed outcome variables was computed by summing all the scores of the items. Following this, all the raw scores were then standardised on a scale of 0–100 to allow for comparisons and interpretability. Higher scores showed better adherence, while lower scores indicated poorer adherence. See Supporting File (S3 Table) for more details. The overall adherence level of HCPs regarding quality newborn care was categorised as poor, moderate, and good. Adherence was considered poor if the HCP obtained an overall score between 0 and 49%. HCPs who obtained a total score of 50–79% were considered moderately adherent, and those who scored between 80 and 100% were considered good adherence. See Fig. 5 for the detailed presentation of the overall level of adherence regarding quality newborn care.

Binary regression was conducted for both bivariate and multivariate analyses to determine the association between independent and dependent variables. A multicollinearity diagnostic test was conducted among the independent variables that were significant for bivariate analyses to determine whether the variables have multicollinearity using the variance inflation factor (VIF). Independent variables with a VIF of more than 10 were not included in the multivariate binary logistic regression model. See details of VIF values as Supporting File (S2 Table). Pairwise correlation was also conducted among dependent variables to determine if the variables were significantly correlated with each other.

## Results

### Socio-demographic characteristics and Individual-level factors

Table 1 presents the demographic characteristics and individual-level factors of participants in this study. Out of 200 participants, 158 agreed to be observed, representing a 79% response rate. Most (63%) were aged between 25 and 31 years. The gender distribution shows a significant majority of females, 111 (70%). About 83 (53%) held bachelor’s degrees, with professional nurses comprising the majority of the sample, at 126 (79%). Work experience among healthcare professionals (HCPs) varied, with most having less than five years of work experience, 87 (55%), and 125 (79%) having worked less than five years in the NICU before data collection. Training on essential newborn care practices (ENCP) was widespread (98%), but a substantial majority, 97 (64%), received ENCP training less than six months prior to data collection. These variables have been published in the study by [27].

### Institutional/Facility Level Factors

Table 2 highlights the institution/facility-level factors in this study. The majority of HCPs, 99 (63%), worked in tertiary-level facilities, with 125 (79%) working in government-owned facilities. A significant majority, 148 (94%), indicated that their facilities organised ENCP training, and 138 (87%) received the training along with regular monitoring and supervision, 130 (82%). Additionally, 133 (84%) reported that their facilities had up-to-date newborn care guidelines, and 135 (85%) indicated that the facilities had adequate hand hygiene facilities to prevent infections.

### Healthcare professionals’ adherence to quality newborn care

In Table 3, the level of healthcare professionals’ adherence to quality newborn care was categorised as “adherent” and “not adherent". The results showed that, under IPC, the majority (84%) adhered to standard guidelines for disposing of medical waste and sharps, while 56% adhered to the guidelines for cleaning medical instruments before use on newborns in the NICU. Under ECEN, 95% correctly assessed and recorded newborns’ breathing rates, and 94% administered and accurately recorded all medications and infusions. Additionally, about 74% effectively communicated the babies’ medical information to caregivers, and 70% of HCPs demonstrated respect toward the babies and their mothers.

### Healthcare professionals’ adherence to Quality Infection Prevention and Control measures

Fig. 2 presents cross-tabulation results on HCPs’ adherence to IPC measures according to professional categories. The analysis reveals that adherence to IPC measures was generally poor, though compliance with waste and sharps disposal exceeded 80% across professions. Conversely, adherence to wearing personal protective equipment (PPE) was low, with medical practitioners at only 6.3%, and highest among auxiliary nurses at 37.5%.

### Healthcare professionals’ adherence to Quality Essential Care for Every Baby

Fig 3 shows findings regarding HCPs’ adherence to quality newborn care on ECEN. Auxiliary nurses showed 100% adherence in assessing and recording breathing rates and administering and recording medications. High adherence was also observed among medical practitioners for weighing (100%), screening for jaundice (93.8%), and classifying newborns for danger signs (93.8%).

### Healthcare Professionals’ adherence to Quality Respectful Maternal and Newborn Care

Fig 4 displays results related to HCPs’ adherence to respectful maternal and newborn care practices. The data reveal that medical practitioners adhered to effective communication practices (93.8%), followed by professional nurses (74.5%). Professional nurses were more respectful toward caregivers (73.8%) compared to medical practitioners and auxiliary nurses. Regarding caregiver education, medical practitioners provided education more frequently (56.3%), while auxiliary nurses did so least often (12.5%).

### Mean Scores of healthcare professionals’ adherence to quality newborn care practices according to professional Categories

Mean scores, based on a four-point Likert scale, indicate better adherence among those with higher scores. Nurses scored higher (83.10) than medical practitioners (47.59) in IPC adherence (p = 0.001). Professional nurses demonstrated greater adherence in IPC (73.77 vs. 53.59, p = 0.047), ECEN (74.40 vs. 48.66, p = 0.010), and respectful maternal and newborn care (74.19 vs. 50.31, p = 0.017). Lastly, healthcare professionals with more than five years of experience had significantly higher mean scores for IPC (91.31 vs. 69.86, p = 0.002), ECEN (86.89 vs. 73.47, p = 0.043), and respectful care (93.18 vs. 68.34, p = 0.000). See the detailed comparison of mean scores in Supporting File (S3 Table).

### Bivariate and Multivariate Logistic regression analysis of HCPs’ adherence to IPC quality standards

The bivariate analyses show that professional nurses [cOR=3.089702 (95% CI: 1.271605, 7.507253), p = 0.013], healthcare providers (HCPs) with a bachelor’s degree [cOR=2.64628 (95% CI: 1.062582, 6.590362), p = 0.037], HCPs with a master’s degree or specialisation [cOR=5.107978 (95% CI: 1.358948, 19.19974), p = 0.016], and HCPs with more than 5 years of work experience [cOR=2.768702 (95% CI: 1.569413, 4.884447), p = 0.000] were 3, 2, 5, and 2 times more likely to follow IPC measures than their counterparts. However, after controlling for covariates, only those with more than 5 years of work experience [aOR=2.243542 (95% CI: 1.160499, 4.33734), p = 0.016] were significantly associated with higher odds of adhering to IPC measures. See detailed results in Table 4 for the bivariate and multivariate results.

**Table 4:** Bivariate and multivariate logistic regression analyses on the factors associated with healthcare professionals’ adherence to IPC guidelines.

Variable	cOR (95% CI), p-value	aOR (95% CI), p-value
**Professional category**		
Auxiliary	Ref.	Ref
Professional nurses	3.089702 (1.271605 7.507253), **0.013**	3.3357 (.8284658 13.43072), 0.090
Medical practitioners	.8311873 (.2462337 2.805759), 0.766	1.227022 (.2315176 6.503101), 0.810
**Professional Qualification**		
Certificate	Ref.	Ref
Diploma	2.270984 (.8747957, 5.895514), 0.092	.8110906 (.231361 2.843469), 0.744
BA/BSc/BEd	2.64628 (1.062582 6.590362), **0.037**	.8807906 (.2792818–2.777811), 0.829
Master’s/Membership/Fellowship	5.107978 (1.358948 19.19974), **0.016**	
**Number of years of work experience**		
Less than 5 years	Ref.	Ref
More than 5 years	2.768702 (1.569413 4.884447), **0.000**	2.243542 (1.160499 4.33734), **0.016**

### Bivariate and multivariate logistic regression analysis of healthcare professionals’ adherence to guidelines on Essential Care for Every Baby

The bivariate analysis showed that professional nurses [cOR=3.183097 (95% CI: 1.265462, 8.006645), p = 0.014] and medical practitioners [cOR=5.03826 (95% CI: 1.53179, 16.57151), p = 0.008] were three and five times more likely, respectively, to adhere to ECEN compared to auxiliary nurses. Again, HCPs with diploma degrees [cOR=3.366173 (95% CI: 1.243653, 9.111161), p = 0.017] and those with bachelor’s degrees [cOR=3.351136 (95% CI: 1.293782, 8.680064), p = 0.013] were significantly more likely to adhere to ECEN compared to HCPs with certificate qualifications or master’s or specialised training. Conversely, HCPs who reported that their facilities organised ENC training were 70% less likely to adhere to essential care [cOR=0.3467922 (95% CI: 1212914, 9915363), p = 0.048]. In multivariate regression analyses, medical practitioners are nearly five times more likely to adhere to ECEN compared to auxiliary and professional nurses [aOR=5.884447 (95% CI: 1.189054, 29.12122), p = 0.030]. See Table 5 for detailed results of the bivariate and multivariate analyses.

**Table 5:** Ordered logistic regression analyses on the factors associated with healthcare professionals’ adherence to Essential Care for Every Newborn.

Variable	cOR (95% CI), p-value	aOR (95% CI), p-value
**Professional category**		
Auxiliary	Ref.	Ref
Professional nurses	3.183097 (1.265462, 8.006645), **0.014**	3.355238 (.9169071 12.27782), 0.067
Medical practitioners	5.03826 (1.53179, 16.57151), **0.008**	5.884447 (1.189054, 29.12122), **0.030**
**Professional Qualification**		
Certificate	Ref.	Ref
Diploma	3.366173 (1.243653, 9.111161), **0.017**	.9512582 (.3247306 2.786593), 0.927
BA/BSc/BEd	3.351136 (1.293782, 8.680064), **0.013**	.8207226 (.2853384 2.360655), 0.714
Master’s/Membership/Fellowship	3.25664 (.8968143, 11.82597), 0.073	
**The health facility organised training on ENCP**		
No	Ref	Ref
Yes	.3467922 (.1212914, .9915363), **0.048**	.3693917 (.1278562, 1.067216), 0.066

### Bivariate and Multivariate Logistic regression analysis of healthcare professionals’ adherence to guidelines on Respectful Maternal and Newborn care

Bivariate analyses revealed that HCPs aged between 32–37 years [cOR=2.029252 (95% CI: 1.03389, 3.982881), p = 0.040] and those between 38 and 44 years [cOR=4.564418 (95% CI: 2.014879, 10.34004), p = 0.000] were significantly more likely to engage in respectful maternal and newborn care practices compared to those aged 25–31 and below. Again, professional nurses [cOR=2.703763 (95% CI: 1.093233, 6.686894), p = 0.031] and those with a bachelor’s degree [cOR=2.720883 (95% CI: 1.070997, 6.912443), p = 0.035] were twice as likely to adhere to respectful maternal and newborn care than their counterparts. Furthermore, HCPs with more than 5 years of work experience, those who are married, nurses at the rank of senior nursing officer, and HCPs who reported the availability of up-to-date guidelines and medical equipment were significantly more likely to adhere to respectful maternal and newborn care [cOR=2.510277 (95% CI: 1.430504, 4.405084), p = 0.001], [cOR=2.296112 (95% CI: 1.315701, 4.007088), p = 0.003], [cOR=3.815423 (95% CI: 1.347204, 10.80568), p = 0.012], [cOR=3.111316 (95% CI: 1.449933, 6.67637), p = 0.004], and [cOR=2.163751 (95% CI: 1.207446, 3.877456), p = 0.010]. Conversely, HCPs who reported that their facilities organised ENC training, those who were Christians, and healthcare facilities at the tertiary level were less likely to adhere to respectful care [cOR=2996439 (95% CI: 0978684, 9174205), p = 0.035], [cOR=4334431 (95% CI: 2009254, 9350384), p = 0.033], and [cOR=22857 (95% CI: 0815781, 6404205), p = 0.005], respectively. In the multivariate analyses, HCPs with more than 5 years of work experience were significantly more likely to adhere to respectful maternal and newborn care than those with less than 5 years of experience [aOR=1.056243 (95% CI: 0379053, 2.074581), p = 0.042]. See Table 6 for detailed information on the bivariate and multivariate analyses.

**Table 6:** Ordered logistic regression analyses on the factors associated with healthcare professionals’ adherence to respectful maternal and newborn care.

Variable	cOR (95% CI), p-value	aOR (95% CI), p-value
**Participants’ age in ranges**		
25-31	Ref.	Ref
32-37	2.029252 (1.03389, 3.982881), **0.040**	.0445606 (−1.047143, 1.136265), 0.936
38-44	4.564418 (2.014879, 10.34004), **0.000**	.3892978 (−1.08551, 1.864106), 0.605
**Professional category**		
Auxiliary	Ref.	
Professional nurses	2.703763 (1.093233, 6.686894), **0.031**	
Medical practitioners	2.972412 (.917488, 9.629811), 0.069	
**Professional Qualification**		
Certificate	Ref.	Ref
Diploma	2.622653 (.9730841, 7.068565), 0.057	
BA/BSc/BEd	2.720883 (1.070997, 6.912443), **0.035**	−.9580395 (−1.980144, .064065), 0.066
Master’s/Membership/Fellowship	3.253744 (.935808, 11.31306), 0.064	−1.601054 (−3.144036, −.058072), **0.042**
**Number of years of work experience**		
Less than 5 years	Ref.	Ref.
More than 5 years	2.510277 (1.430504, 4.405084), **0.001**	1.056243 (.0379053, 2.074581), **0.042**
**Facility organised training on ENCP**		
No	Ref.	Ref
Yes	.2996439 (.0978684, .9174205), **0.035**	−1.489895 (−2.923535, −.0562551), 0.042
**Marital Status**		
Single	Ref.	Ref
Married	2.296112 (1.315701, 4.007088), **0.003**	.6429407 (−.0814763, 1.367358), 0.082
**Religion**		
Islam	Ref.	Ref
Christianity	.4334431 (.2009254, .9350384), **0.033**	−.5453584 (−1.479646, .3889291), 0.253
**Rank (Professional Nurse)**		
Staff nurse	Ref.	Ref
Senior staff nurse/Senior staff midwife	1.687137 (.6140937, 4.635177), 0.310	
Nursing/midwifery Officer	.9145031 (.3820886, 2.188801), 0.841	
Senior nursing/midwifery Officer	3.815423 (1.347204, 10.80568), **0.012**	1.291057 (−.3568628, 2.938977), 0.125
Principal Nursing/Midwifery Officer	2.510999 (.714177, 8.828502), 0.151	
**Level of facility**		
Primary	Ref.	Ref
Secondary	.3797675 (.1296126, 1.112727), 0.078	
Tertiary	.22857 (.0815781, .6404205), **0.005**	−1.099832 (−2.407319, .2076546), 0.099
**Availability of up-to-date newborn care guidelines**		
No	Ref.	
Yes	3.111316 (1.449933, 6.67637), **0.004**	.7474362 (−.1643607 1.659233), 0.108
**Availability of medical equipment at NICU**		
No	Ref.	Ref
Yes	2.163751 (1.207446, 3.877456), **0.010**	.3564154 (−.3691634, 1.0819940, 0.336

### Overall level of adherence to quality newborn care practices among healthcare professionals

Fig. 5 highlights the overall HCP adherence to IPC, essential care for every newborn, and respectful maternal and newborn care. Overall, 20% of HCPs demonstrated good adherence to quality newborn care practices, while 46% showed poor adherence. Regarding ECEN and respectful maternal and newborn care, 53% and 51% of HCPs demonstrated moderate adherence, respectively.

### Responses from HCPs during facility-level validation of research findings

#### Adherence to hand hygiene practices.

This section presents responses from the observed healthcare professionals during the facility-level validation of the study findings. In this section, three key areas of interest were discussed, and the participants responded to the findings. These areas were infection prevention and control (IPC), umbilical cord care (chlorhexidine use), and management of neonatal pain.

#### Infection prevention and control (IPC).

On the aspect of IPC, the study revealed that the majority (85%) of the HCPs indicated that their facilities had adequate hand hygiene facilities in the NICUs for infection prevention and control. However, the observations showed that adherence to IPC was poor, as the participants did not adhere to the standard hand hygiene practices as recommended by WHO and GHS. The participants indicated factors such as the wrongful placement of handwashing sinks, irregular water flow, inadequate supply of methylated spirit, and increased workload due to inadequate staffing. For instance, one HCP explained the shortage of handwashing sinks and irregular water flow:

“*The results represent what happens in our NICU. This NICU comprises four cubicles with only one handwashing sink in the unit with limited water flow. With this, it becomes difficult to practise effective hand hygiene even if you want to.”*

Another HCP explained how staff shortage prevented them from practising proper hand hygiene:

*“I agree with your observation. In our NICU here the pressure is too much on us, and we are just very few always on duty. Even if you want to practise proper hand hygiene using all these protocols, you will not be able to do that because of the pressure on you. So, we just do anything in the name of handwashing so we can have time to care for our babies.”*

However, one of the wards’ in-charges felt that, despite these challenges, the decision not to practise IPC measures is based on the individual. This is what he had to say.

*“I agree with the observed findings. I believe this is on an individual basis. As you observed, all the IPC protocols are in the ward, but it is left to the individual to practise the right things. We have been encouraging them to practise effective hand hygiene because the data show that our babies are dying a lot in the ward.”*

#### Umbilical cord care (chlorhexidine use).

The findings also revealed that the majority (86%) of the HCPs did not apply chlorhexidine gel to the babies’ umbilical cords as recommended by WHO and GHS. They attributed this to parental preference for methylated spirit instead of chlorhexidine, poor environmental conditions in NICUs (cold environments), and lack of chlorhexidine in the NICUs for at least one week. One of the HCPs explained how the environmental condition in the NICU affects how the umbilical cord is cared for. This is what he had to say:

“*We do not apply chlorhexidine to the babies’ umbilical cords in the NICU because the chlorhexidine is water-based, which keeps the cord moist. Our NICU is always cold, so applying the chlorhexidine to the cord in the cold NICU will keep the cords moist, leading to smelly cords, and this can cause cord infections”.*

Another HCP elaborated on how the lack of chlorhexidine in the NICU and parental preference of cord care affect proper umbilical cord practices. This is what she had to say:

“*Our NICU doesn’t have chlorhexidine for the newborns. We rely on the caregivers of babies to buy the chlorhexidine for use. But they complained it is costly. Some parents also complained that the chlorhexidine makes the cord wet, prolonging the detachment period, and that we should not apply it on their newborns’ cords.” “We explained the importance of chlorhexidine as the recommended gel to be applied on the umbilical cord to the caregivers”.*

#### Adherence to neonatal pain management.

It was also observed that almost all (87%) of the HCPs did not assess and manage newborn pain using an appropriate pain assessment scale. They attributed this to increased workload and lack of pain assessment tools and protocols, among others. For instance, one of the healthcare professionals explained how a busy work environment and a lack of pain assessment tools result in poor pain assessment and management. The HCPs had this to say:

*“The NICU is a busy place with a lot of sick babies, and you don’t have time to even rest. So, it is difficult to assess their pain. As you observed, there are no pain assessment protocols in the NICU, so we don’t assess their pain levels.”*

Another HCPs indicated:

*“The main problem is that our workload here is high, so we don’t have the time to monitor the babies’ pain. Aside from this, it is even difficult to know when the babies are in pain, so you can monitor them. Any touch on them results in crying. When this happens, we just call their mothers to pick them up.”*

## Discussion

The study evaluated how well clinical healthcare professionals in neonatal intensive care units across Ghana follow quality newborn care practices.

Observations under infection prevention and control (IPC) revealed poor adherence to handwashing and alcohol hand rub guidelines, despite healthcare professionals (HCPs) reporting ample availability of hand hygiene facilities. The study also found serious lapses in intravenous catheter care during insertion, maintenance, and access. During the validation session, healthcare professionals attributed these lapses to factors such as incorrect placement of handwashing sinks, inconsistent water flow, insufficient supplies of methylated spirit, and increased workload due to staffing shortages. Regarding essential care for every newborn, the HCPs demonstrated high adherence to monitoring vital signs like temperature and respiration and examining for jaundice and danger signs. However, adherence was poor for pain assessment and umbilical cord care. The validation session revealed that these gaps were caused by busy ward schedules, increased workload, and a lack of pain assessment protocols. Caregivers’ involvement in decision-making was limited, resulting in fewer receiving education and information about their newborns’ health. Nonetheless, most HCPs communicated effectively with caregivers, respecting their dignity, privacy, and confidentiality.

Effective infection prevention by HCPs is vital for health outcomes, but evidence shows low compliance with IPC measures in healthcare settings. Globally, in 2018, the IPC compliance level in intensive care units was 59.6% [36]. Poor hand hygiene and catheter care practices can lead to healthcare-associated infections (HAIs) and increased neonatal mortality, with intensive care mortality rates rising from 24.4% to 52.3% globally due to HAIs [37]. Our findings align with a similar observational study in Pakistan, which reported 48.9% adherence to hand hygiene practices among HCPs [38], 30% in Nepal [39], 29.1% in Nigeria [40] and 51% in Ghana [41]. Higher compliance has been reported in Ghana [42]. These findings highlight a significant gap in infection prevention in Ghanaian NICUs. Poor adherence to IPC increases the risk of pathogen transmission among neonates. Failing to follow aseptic intravenous catheter care procedures raises the chance of bloodstream infections, sepsis, longer hospital stays, and neonatal death [43, 44]. The WHO recommends training clinicians on hand hygiene and aseptic “no touch” techniques and regular assessments of knowledge and adherence to prevent catheter-related infections, including among neonates [45]. As healthcare professionals noted in validation sessions, logistical challenges, staff attitudes, and workload contribute to poor IPC adherence. Training alone, without providing the necessary supplies, is unlikely to improve practices. Increased investment in facilities is crucial. Ongoing professional development and the appointment of IPC champions in NICUs, who can serve as role models, may be key to improving infection prevention. The government, through agencies like the Ghana Health Service and the Ministry of Health, should invest more in IPC measures to prevent HAIs and improve neonatal outcomes. Healthcare workers with over five years of experience were more likely to adhere to IPC protocols, possibly due to greater familiarity, training, and a sense of responsibility, which are supported by previous studies [46, 47].

Regarding essential care for every newborn, monitoring and recording vital signs showed consistency with previous research [38, 48], as this practice is critical for detecting issues such as hypothermia, hyperthermia, and respiratory problems like apnoea and respiratory distress syndrome, which are common in small and sick newborns characterised by breathing difficulties, nasal flaring, grunting, and chest indrawing [49]. Prompt respiratory support and regular temperature checks are essential to manage these potential complications. Most HCPs in this study reported receiving regular training on ENCP, monitoring and supervision from their superiors, and resource availability. These factors could have led to increased adherence to clinical guidelines, institutional policies on newborn care, clinical experience, and professional responsibility. At the NICUs, clinical staff are expected to assess and manage neonates’ pain [50, 51]. However, adherence to pain assessment and management was poor, contradicting WHO recommendations. Failure to adequately assess and treat pain can lead to undetected suffering, resulting in severe physiological problems and impacting overall health [49]. During validation discussions, HCPs cited misconceptions that neonates do not feel pain, increased workload, lack of time, and absence of pain scales as barriers. These factors are consistent with previous research findings [52]. One study also pointed out that cultural beliefs influence pain management, though this was not observed in our study [53]. The American Pain Society regards pain as the fifth vital sign, emphasising its importance in care [54]. We urge healthcare facility leaders to develop and implement pain assessment guidelines for routine practice.

Concerning umbilical cord care, poor adherence by HCPs to examining the cord and applying chlorhexidine, as recommended by WHO and GHS, fosters unhygienic practices and raises the risk of infections that can be fatal. Chlorhexidine significantly reduces neonatal sepsis and mortality [55, 56]. Barriers to effective cord care mentioned by HCPs during validation included shortages of chlorhexidine gel, its unsuitability in cold NICU environments, and parental preference for methylated spirits over chlorhexidine, often due to misconceptions that it delays umbilical cord detachment time. These factors revolve around institution-based challenges and healthcare professional and caregiver misconceptions. We call on the Ghana Health Service and the Ministry of Health to increase awareness about chlorhexidine’s role in preventing neonatal infections, dispelling misconceptions among HCPs and caregivers, and improving knowledge on its application. When NICU staff do not adhere to applying chlorhexidine, they may also not educate and encourage caregivers to apply it at home. This may give rise to unhygienic home umbilical cord practices by caregivers. Therefore, ensuring adequate supplies of chlorhexidine in NICUs and maternity wards is timely. The study found that medical practitioners were five times more likely to follow essential care practices than nurses, possibly due to differences in training, roles, and autonomy. Addressing these disparities in adherence level is vital to ensure consistent newborn care and better outcomes for small and sick infants.

Our results on respectful maternal and newborn care mirror previous studies in Namibia and Italy that used WHO quality standard statements, showing that most healthcare professionals communicated effectively and respected caregiver dignity [30–32]. Despite effective communication between caregivers and HCPs in these previous studies, there were reports of disrespect leading to caregivers’ refusal of treatment. Similar findings of effective communication and respectful care were observed in our study. However, fewer HCPs involved caregivers in decision-making in this study, leading to ineffective education and information sharing on babies’ health. Our findings are incongruent with previous studies, where many were actively involved in the care process [30, 32]. Engaging caregivers promotes empowerment, enhances communication, decision-making, bonding, reduces anxiety, decreases readmissions, and supports breastfeeding and weight gain [57, 58]. Inadequate health education may cause misunderstandings and reduce caregivers’ capacity to care for their babies, potentially affecting health-seeking behaviour [59].

## Limitations and strengths

This study has limitations: healthcare professionals might have altered their behaviours if aware they were observed (Hawthorne effect), possibly overestimating adherence. Despite using structured checklists, observer bias could occur, especially with subjective judgements. Though the study was conducted in three main geographic belts of Ghana, it may not fully capture the diversity of healthcare practices across the country, especially in rural areas.

Nonetheless, the study demonstrated enormous strengths. It was conducted among health facilities categorised into primary, secondary, and tertiary levels. This provided a broader view of adherence across facilities necessary for policy decision-making. Also, the use of an observational approach allowed for a direct examination of HCPs’ actual care behaviours, thereby reducing bias compared to self-reporting. Using two independent observers per site further improved reliability and reduced observer bias. Furthermore, addressing various aspects of newborn care provides comprehensive, evidence-based insights essential for developing country-specific and locally tailored interventions, policy reforms, and training opportunities to improve neonatal outcomes.

## Conclusion and recommendations

Overall, the study identified major gaps in adherence to newborn care guidelines in Ghana. While practices like vital sign monitoring and medication management were well followed, infection prevention measures such as hand hygiene, aseptic techniques, pain assessment, and umbilical cord care were poorly adhered to. Caregiver involvement, especially in decision-making, was limited, with less than half of caregivers receiving health education on their newborns’ health. These findings emphasise the urgent need to improve compliance with IPC, pain management, cord care, and caregiver engagement, crucial steps toward better neonatal health and progress toward SDG 3.2 attainment.

Therefore, we recommend that health facilities ensure consistent availability of hand hygiene equipment (soap, veronica buckets, sinks, alcohol, among others) within the immediate working environment of HCPs. They should also ensure facility-level IPC audits and organise regular training on updated guidelines on IPC. Furthermore, the NICUs should promote family-centred care and train healthcare professionals in respectful maternal and newborn care. This will empower and enable caregivers and promote an inclusive caring environment that fosters shared decision-making during the care process, as endorsed by WHO and UNICEF. The facilities should strengthen quality assurance interventions, including tracking key quality indicators in newborn care, such as caregiver education and involvement, hygiene compliance, discharge educations/interviews, and making available suggestion boxes, among others. Lastly, policymakers in the Ghana Health Service, including the institutional care division and family health division, should incorporate these findings into strengthening existing strategies to ensure quality newborn care and improve survival rates.

## Supporting information

S1 File STROBE Checklist. (DOCX)

S1 Table WHO Standards. (DOCX)

S2 Table Variance Inflation Factor (VIF). (DOCX)

S3 Table Mann-Whitney Test Values. (DOCX)

## Abbreviations

WHO: World Health Organization
GHS: Ghana Health Service
SSA: sub-Saharan Africa
NM: Neonatal Mortality
NMR: Neonatal Mortality Rate
UN SDGs: United Nations Sustainable Development Goals
ENAP: Every Newborn Action Plan
QoC: Quality of Care
HCP: Healthcare Professionals
ECEN: Essential Care for Every Newborn
NICU: Neonatal Intensive Care Units
IPC: Infection Prevention and Control
VIF: Variance Inflation Factor
TFR: Total Fertility Rate
NHIA: National Health Insurance Authority
FMHCP: Free Maternal Healthcare Policy
HeFRA: Health Facilities Regulation Authority
CEmONC: Comprehensive Emergency Obstetric and Newborn Care
STROBE: Strengthening Reporting of Observational Studies in Epidemiology
UNICEF: United Nations Children’s Fund
