# Supplementary material for: Benchmarking newborn care quality in Ghana: Evidence from structured observations of clinical practice against WHO quality standards
Source: PLoS One. 2026 Jun 11;21(6):e0350931. doi: 10.1371/journal.pone.0350931 (PMC13257978; doi:10.1371/journal.pone.0350931)
Supplement: S1 Table — (DOCX) [file pone.0350931.s002.docx]

**Supporting File 2: Details of WHO quality of care standards and Quality Statements**

| **STANDARDS** | **QUALITY STATEMENTS** |
| --- | --- |
| **STANDARD 1:** Every small and sick newborn receives evidence-based routine care and management of complications | 1. All newborns receive care with standard precautions to prevent healthcare-associated infections |
|  | 1. All newborns are assessed immediately while receiving essential newborn care |
|  | 1. All newborns receive routine postnatal care, including weighing and temperature measurement |
|  | 1. All newborns are assessed for suspected infection or risk factors for infection and, if required, investigated and given the correct antibiotic treatment |
|  | 1. Small and sick newborns are assessed for signs of respiratory compromise, and a neonatal pulse oximeter is used to detect hypoxia or hyperoxia and to guide administration of supplemental oxygen |
|  | 1. Small and sick newborns who require supplemental oxygen therapy receive it safely through appropriate neonatal equipment, including neonatal nasal prongs, low-flow meters, air–oxygen blenders, humidifiers and a pulse oximeter |
|  | 1. Small and sick newborns are assessed and managed for apnoea, and preterm newborns are managed to prevent apnoea |
|  | 1. Newborns with respiratory distress are treated with continuous positive airway pressure as soon as the diagnosis is made |
|  | 1. Small and sick newborns are fed appropriately, including assisted feeding with the mother’s milk when possible |
|  | 1. All newborns are routinely monitored for jaundice; bilirubin is measured in those at risk and treatment initiated in those with hyperbilirubinaemia |
|  | 1. Small and sick newborns are assessed and managed for seizures |
|  | 1. Small and sick newborns, especially those who are most seriously ill, are adequately monitored, appropriately reassessed and receive supportive care |
|  | 1. Small and sick newborns are given antibiotics and other medications only if indicated, by the correct route and of the correct composition; the dose is calculated, checked and administered, the need for medication is regularly reassessed, and any adverse reaction is appropriately managed and recorded |
|  | 1. Small and sick newborns who cannot tolerate full enteral feeds are given intravenous fluids containing glucose or safe, appropriate parenteral nutrition; fluids are administered through an infusion pump and a neonatal burette, the volume is recorded, and the intravenous site is checked with other routine observations |
|  | 1. All small and sick newborns are assessed routinely for pain or symptoms of distress and receive appropriate management |
| **STANDARD 4:** Effective Communication, Meaningful participation for caregivers and newborns, and Parental involvement in care | 1. All carers of small and sick newborns are given information about the newborn’s illness and care, so that they understand the condition and the necessary treatment |
|  | 1. All small and sick newborns and their carers experience coordinated care, with clear, accurate information exchange among relevant health and social care professionals and other staff |
|  | 1. All carers are enabled to participate actively in the newborn’s care through family-centred care and kangaroo mother care, in decision-making, in exercising the right to informed consent and in making choices. |
|  | 1. All carers receive appropriate counselling and health education about the current illness of the newborn, transition to kangaroo mother care follow-up, community care and continuous care, including early intervention and developmental follow-up |
| **STANDARD 5:** Newborns’ rights are respected, protected and fulfilled without discrimination, with preservation of dignity at all times and in all settings during care | 1. All newborns have equitable access to health care services, with no discrimination of any kind |
|  | 1. The carers of all newborns are made aware of and given information about the newborn’s rights to health and health care |
|  | 1. All newborns and their carers are treated with respect and dignity, and their right to privacy and confidentiality is respected |
