# Supplementary material for: Benchmarking newborn care quality in Ghana: Evidence from structured observations of clinical practice against WHO quality standards
Source: PLoS One. 2026 Jun 11;21(6):e0350931. doi: 10.1371/journal.pone.0350931 (PMC13257978; doi:10.1371/journal.pone.0350931)
Supplement: S2 Table — (DOCX) [file pone.0350931.s003.docx]

**Supporting File 4: Mean scores showing adherence to quality newborn care practices**

|  | **Professional category** | |  |  |  |
| --- | --- | --- | --- | --- | --- |
| **Variables** | **Nurses**  **Mean** | **Medical professionals**  **Mean** | **Mann Whitney** | **Z** | **Significance level** |
| Infection Prevention and Control | 83.10 | 47.59 | 625.500 | 3.184 | **0.001** |
| Essential care for every newborn | 79.24 | 81.81 | 1099.000 | .235 | 0.814 |
| Respectful maternal and newborn care | 79.26 | 81.66 | 1101.500 | .217 | 0.828 |
|  | **Professional and Auxiliary Nurses** | |  |  |  |
| **Variables** | **Auxiliary Nurses**  **Mean** | **Professional Nurses**  **Mean** | **Mann Whitney** | **Z** | **p-values** |
| Infection Prevention and Control | 53.59 | 73.77 | 721.500 | 1.985 | **0.047** |
| Essential care for every newborn | 48.66 | 74.40 | 642.500 | 2.566 | **0.010** |
| Respectful maternal and newborn care | 50.31 | 74.19 | 669.500 | 2.381 | **0.017** |
|  | **Number of Years of Working Experience** | |  |  |  |
| **Variable** | **Less than 5 years** | **5 years and above** | **Mann Whitney** | **Z** | **p-value** |
| Infection Prevention and Control | 69.86 | 91.31 | 2250.000 | 3.172 | **0.002** |
| Essential care for every newborn | 73.47 | 86.89 | 2563.500 | 2.024 | **0.043** |
| Respectful maternal and newborn care | 68.34 | 93.18 | 2117.500 | 3.703 | **0.000** |

***Source: Field data (2023-2024)***

Legend: *Mann-Whitney U test, a two-tailed test of means with statistical significance at a 95% confidence level. The Mean values show adherence from the summated four-point Likert scale. Higher values show better adherence, while lower values show less adherence to quality newborn care practices.
