# Supplementary material for: Benchmarking newborn care quality in Ghana: Evidence from structured observations of clinical practice against WHO quality standards
Source: PLoS One. 2026 Jun 11;21(6):e0350931. doi: 10.1371/journal.pone.0350931 (PMC13257978; doi:10.1371/journal.pone.0350931)
Supplement: S3 Table — (DOCX) [file pone.0350931.s004.docx]

**Supporting File 3: Variance Inflation Factor Values**

| **IPC** | | **Essential care for every baby** | | **Respectful maternal and newborn care** | |
| --- | --- | --- | --- | --- | --- |
| **Variables** | **VIF** | **Variables** | **VIF** | **Variables** | **VIF** |
| Professional category | 1.85 | Professional category | 1.52 | Marital status | 1.28 |
| Professional qualification | 2.11 | Professional qualification | 1.51 | Religion | 1.15 |
| Number of years of working experience (more than five years) | 1.41 | Facility organized training on ENCP | 1.00 | Participants age in range | 2.11 |
|  |  |  |  | Professional category | 1.40 |
|  |  |  |  | Professional qualification | 2.17 |
|  |  |  |  | Professional rank (nursing) | 2.65 |
|  |  |  |  | Number of years of working experience (more than five years) | 2.15 |
|  |  |  |  | Level of facility | 1.18 |
|  |  |  |  | Availability of up-to-date guidelines | 1.16 |
|  |  |  |  | Availability of medical equipment | 1.20 |
|  |  |  |  | Facility organized training on ENCP | 1.20 |

Legend: VIF=Variance Inflation Factor; ENCP= Essential Newborn Care Practices; IPC= Infection Prevention and Control
